# Predictors of organizational commitment among university nursing Faculty of Kathmandu Valley, Nepal

**DOI:** 10.1186/s12912-018-0298-7

**Published:** 2018-07-16

**Authors:** Rekha Timalsina, Sarala K.C., Nilam Rai, Anita Chhantyal

**Affiliations:** 1 Patan Academy of Health Sciences, School of Nursing and Midwifery, Lalitpur Nursing Campus, Lalitpur, Nepal; 2 Dristi Nepal, Kathmandu, Nepal; 3 Tribhuvan University Teaching Hospital, Maharajgunj, Kathmandu, Nepal

**Keywords:** Nursing faculty, organizational commitment, Predictors

## Abstract

**Background:**

Increasing work efficiency, improving psychological health, decreasing turnover, turnover intention, and absenteeism may be dependent on organizational commitment of an employee. This study was carried out to identify the predictors of organizational commitment among university nursing faculty within Kathmandu Valley, Nepal.

**Methods:**

A cross-sectional analytical study was conducted based on a sample of 197 nursing faculty selected from 18 nursing colleges affiliated to 5 universities in Kathmandu Valley by using a proportionate stratified random sampling technique. Structured questionnaires regarding socio-demographic information, perceived faculty developmental opportunity, job satisfaction, perceived organizational support, and organizational commitment were used for data collection. Double data entry and data cleaning were done by using Epi-data software; and data analysis was carried out with SPSS version 16 software. Binary regression analysis was used to identify the predictors of organizational commitment and the adjusted odds ratio (AOR) was also calculated.

**Results:**

The findings of this study showed that a majority of respondents had moderate level of organizational commitment (68%) followed by high level (29%) and low level (3%). This study also revealed that the nursing faculty who had a master’s degree in nursing, a permanent appointment, and job satisfaction had a high level of organizational commitment. On the contrary, this study also revealed that the nursing faculty who were in the position of assistant instructor to assistant lecturer level and more than 5 years of work experience within same organization were less likely to have a high level of organizational commitment.

**Conclusions:**

Nursing faculty within Kathmandu Valley have a moderate level of organizational commitment. The predictors of organizational commitment are higher education in nursing, position, type of appointment, current organizational tenure, and job satisfaction. Therefore, an organizational authority must pay attention to the modifiable predictors of organizational commitment to enhance organizational commitment of its nursing faculty. This will help to reduce faculty turnover, increase quality of teaching and student’s satisfaction.

## Background

Organizational commitment refers to employee commitment to an organization regarding desire-based (affective commitment), obligation based (normative commitment) and cost-based (continuance commitment) [1]. These form an ecosystem that encourage an employee to voluntarily continue working in an organization [2]. Organizations often try to foster commitment in their employees, which provides an impetus to work harder and be more enterprising to achieve organizational objectives. A combination of these factors ensures that an organization attains stability and reduces costly employee turnover [1]. Therefore, the faculty’s commitment to their organization, students, teaching activities, occupation, and colleagues has a positive influence on the effectiveness of an academic institution [3]. Universities worldwide are aiming to retain committed faculty in their system [4].

Job satisfaction is the most dominant factor in organizational commitment [5] and a previous study on predictors of nursing faculty members’ organizational commitment in governmental universities in Jordan showed that age, job satisfaction, and perceived organizational support were significantly related to faculty members’ commitment [6]. Similarly, ex post-facto type descriptive study of private universities’ faculty and staff in Nigeria highlighted that marital status, job type, and job tenure significantly predicted organizational commitment and turnover intention [7]. A comparative study on qualification and organizational commitment among the faculty of private universities in Pakistan revealed that faculty members with a master’s degree were more dedicated than those with either an MPhil or PhD degree [8]. In Nepal’s context, based on available literature, there has been only one study on factors associated with organizational commitment among nurses. This study revealed that 34% of nurses had a high level of organizational commitment, and perceived organizational support was associated with organizational commitment [9]. A qualified and committed nursing faculty is essential to sustain a nursing institution and deliver high quality education. However, there is no rigorous academic inquiry into organizational commitment among nursing faculty and its associated factors. This study was aimed to determine the predictors of organizational commitment among university nursing faculty within Kathmandu Valley, Nepal so that it can help the nursing administrators and managers of various universities to find ways to improve organizational commitment.

## Methods

### Study design

A cross-sectional analytical study was conducted to identify the predictors of organizational commitment among university nursing faculty of Kathmandu Valley.

### Population and sample

The study population consisted of 279 nursing faculty who had completed at least six months of a full-time teaching in a bachelor program or higher level of 18 nursing colleges affiliated to 5 universities (i.e., Tribhuvan University (TU), Purbanchal University (PU), Pokhara University (PokU), Kathmandu University (KU) and National Academy for Medical Sciences (NAMS) of Kathmandu Valley). But, nursing faculty on long leave were excluded from the study. The campus chief, assistant campus chiefs, heads of departments, and visiting professors were excluded from the study population. The sample size was calculated by using the formula [10]:

$$ \mathbf{n}=\left[\left({\mathrm{z}}^2\mathrm{pq}\right)+\mathrm{ME}2\left]/\right[\mathrm{ME}2+{\mathrm{z}}^2\mathrm{pq}/\mathrm{N}\right] $$

where.

**Z** = 1.96 for 95% confidence level,

***p*** = 68% [3], **q** = 1-p,

ME (Margin of Error) = 5%,

**n** = Sample Size.

**N** = Population Size (i.e., 279).

**Fig. 1:**
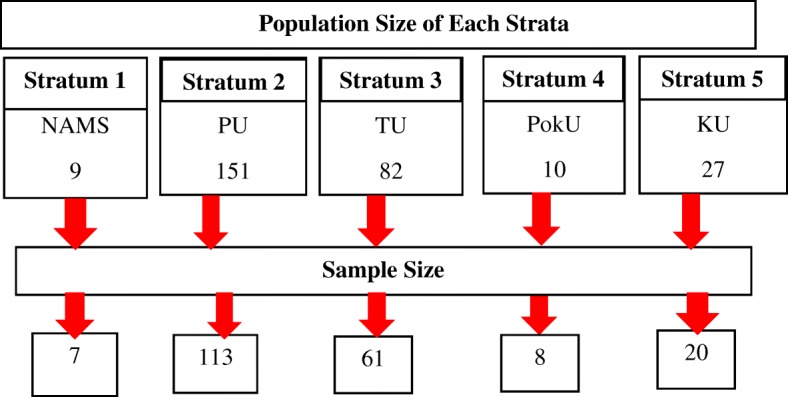
Strata, Population Size and Sample Size of Different Universities

The required sample size was 152 nursing faculty for 95% confidence level. Allowing non-response rate of 10% and maintaining the power of test (i.e., n’/0.8), the final sample size was estimated to be 209 nursing faculty. The proportionate stratified random sampling technique was used to divide the population into 5 strata of affiliated universities (See Figure 1). Sample size for each stratum was determined by using following equation [11]: n_h_ = (N_h_/N) × n^*^, where, n_h_: sample size for stratum *h*, N_h_: population size for stratum *h*, N: total population size, n^*^: total sample size.

After stratification, the sample of 209 nursing faculty were selected with simple random sampling technique from each stratum using random numbers from a random number generator [12].

### Research instruments

The instruments used in this study were composed of five parts. Part one was related to structured questionnaire on socio-demographic and personal information.

Part two consisted of 15 items of perceived faculty developmental opportunities measured on a 5-point Likert scale ranging from 1 (strongly disagree) to 5 (strongly agree) with 5 negatively worded items. The minimum and maximum scores of this scale were 15 and 75 respectively. The perception level was categorized into five categories as Most Favorable Perception (75 score), Favorable Perception (46–74 score), Neutral Perception (45 score), Unfavorable Perception (16–44 score) and Most Unfavorable Perception (15 score) [13].

Part three consisted of 36 items of job satisfaction survey (JSS) of nursing faculty measured on 6-point Likert scale ranging from 1 (Strongly disagree) to 6 (Strongly agree) with 19 negatively worded items. The minimum and maximum scores were 36 and 216 respectively. The level of job satisfaction was determined by the score of respondents. A mean item response ≥4 represented satisfaction, whereas mean response ≤3 represented dissatisfaction, and mean scores between 3 and 4 represented as ambivalence [14].

Part four consisted of 8 items of perceived organizational support (POS) [15] of nursing faculty measured on 7-point Likert scale ranging from 0 (strongly disagree) to 6 (strongly agree) with 4 negatively worded items. The minimum and maximum scores were 0 and 48 respectively. The level of POS was determined by the score of the respondents. The scores with 1 standard deviation above mean represented as high POS, whereas 1 standard deviation below mean represented as low POS [16].

Part five consisted of 18 items of organizational commitment questionnaire (OCQ) - affective, continuance, and normative [1], measured on 7-point Likert scale ranging from 1 (strongly disagree) to 7 (strongly agree) with 4 negatively worded items. The minimum and maximum scores were 18 and 126 respectively. The level of organizational commitment was determined by score of the respondent. Thus, the scores fell in of the following ranges: 1.00–3.00: Low Commitment, 3.01–5.00: Moderate Commitment and 5.01: 7.00: High Commitment [17]. Negatively worded items of these instruments were reversed before analysis.

The OCQ tool has been already validated in the Nepalese context [18]. To ensure content validity for current study, OCQ along with other instruments were further evaluated by experienced professional nursing faculty, human resource managers, and academics in nursing in order to determine if the instrument reflects the known content area. The OCQ, JSS and POS had already been translated and back translated and used in previous study conducted among nurses in 2015 [9]. Reverse translation of perceived faculty developmental opportunity scale was done for assuring semantic equivalence of this instrument. Pretesting of those instruments was done among 22 nursing faculty from three nursing colleges who were similar in characteristics with actual samples of this study and these colleges were excluded in actual study. This was done for establishing the conceptual/linguistic and functional equivalence. Reliability coefficient based on Cronbach alpha was 0.72, 0.70, 0.85 and 0.81 for perceived faculty developmental opportunity, OCQ, JSS and SPOS respectively.

### Data collection procedure

Written permission was taken from the authors for using OCQ and SPOS. JSS is a free tool for educational purposes. Data was collected from 23 January 2017 to 13 March 2017 by applying ethical procedure. The questionnaires were distributed to 209 nursing faculty selected in sample for self-response with provision of sufficient time as requested by the respondents (average 3 days’ time) and regular follow-up. Within time frame of data collection period allocated for the study, 197 nursing faculty returned the completed questionnaire and 12 questionnaires were not returned. Thus, a valid response rate for this study was 94.3% and the data analysis was carried out using 197 completed questionnaires.

Questionnaire editing for completeness and consistency of responses was done at field and central level. Double data entry as well as data cleaning was done using Epi Data software and data analysis was done using SPSS software version 16. Descriptive statistics was used to describe the sample characteristics and binary logistic regression analysis was used to identify the predictors of organizational commitment and adjusted odds ratio was also calculated. Logistic regression adjusted for age, marital status, higher education in nursing, higher education besides nursing, position, type of appointment, positional tenure, professional tenure, current organizational tenure, family structure, perceived faculty developmental opportunity, job satisfaction and perceived organizational support. Variables were entered simultaneously in the model and significant results were denoted by an asterisk in the table. For each test, significance was considered at *p* ≤ .05 for 95% confidence level.

## Results

In this study, the nursing faculty were aged from 24 to 73 years (*M* = 36 years, *SD* = 8.3); 86.8% were married; 54.8% had a master’s degree in nursing; 40.1% received education besides nursing; 49.7% had economically dependent family members; 46.2% had a permanent type of appointment; and 48.7% were lecturers. Majority of respondents had less than five years of positional tenure (76.6%) and organizational tenure (64.5%), and ≥ 9 years of professional tenure (74.1%).

Table 1 reveals that 29% of respondents had a high level of organizational commitment (M = 4.63, SD = 0.71).

**Table 1:** Respondent’s Level of Organizational Commitment

				*n* = 197
Level of Organizational Commitment	Frequency	Percent	Min-Max	M ± SD
Low	6	3		
Moderate	134	68	2.72–6.22	4.63 ± 0.71
High	57	29		

*Note.* Mean scores 1.00–3.00: Low Commitment, Mean scores 3.01–5.00: Moderate Commitment and Mean scores 5.01–7.00: High Commitment [17]

Table 2 shows that a higher education in nursing (*p* = .031, AOR: 3.743, CI: 1.129, 12.410), and type of appointment (*p* = .000, AOR: 4.542, CI: 1.960, 10.525) contributed significantly to the prediction of a high level of organizational commitment, whereas position (*p* = .022, AOR: 0.242, CI: 0.072, 0.816) contributed significantly to the prediction of a low to moderate level of organizational commitment. The contribution of other socio-demographic factors- age, marital status, and higher education besides nursing were not significant. However, the adjusted odds ratio reveals that respondents who were above 30 years old, unmarried, and had higher education besides nursing were 1.861, 1.598 and 1.348 times more likely to have a higher level of organizational commitment respectively. On the other hand, the respondents with position of Assistant Instructor to Assistant Lecturer were less likely to have higher level of organizational commitment than lecturer to Professor.

**Table 2:** Analysis of Respondent’s Socio-demographic Characteristics as a Predictors of Organizational Commitment

					*n* = 197
Socio- demographic Characteristics	Organizational Commitment		*p*-value ^a^	AOR	95% CI
	Low and Moderate Level	High Level			
	*N* (%)	*N* (%)			
Age Group					
Up to 30 years ^b^	48 (82.8)	10 (17.2)			
> 30 years	92 (66.2)	47 (33.8)	.278	1.861	[0.606, 5.714]
Marital Status					
Married^b^	22 (84.6)	4 (15.4)			
Unmarried	118 (69.0)	53 (31.0)	.482	1.598	[0.432, 5.905]
Higher Education in Nursing					
Bachelor ^b^	67 (75.3)	22 (24.7)			
Master	73 (67.6)	35 (32.4)	.031^c^	3.743	[1.129, 12.410]
Higher Education Besides Nursing					
No^b^	88 (74.6)	30 (25.4)			
Yes	52 (65.8)	27 (34.2)	.465	1.348	[0.605, 3.004]
Position					
Assistant Instructor to Assistant Lecturer	54 (70.1)	23 (29.9)	.022^c^	0.242	[0.072, 0.816]
Lecturer to Professor Level^b^	86 (71.7)	34 (28.3)			
Type of Appointment					
Temporary/Contract ^b^	89 (84.0)	17 (16.0)			
Permanent	51 (56.0)	40 (44.0)	.000^c^	4.542	[1.960, 10.525]

*Note.* ^a^: *p*-value obtained from binary logistic regression. ^b^ = reference category. ^c^ = *p*-value significant at ≤0.05 level. *AOR* Adjusted Odds Ratio

Table 3 highlights that current organizational tenure (*p* = .044, AOR = 0.039, CI = 0.098, 0.967) contributed significantly to the prediction of a low to moderate level of organizational commitment, but, positional tenure and professional tenure did not. However, the adjusted odds ratio reveals that respondents with greater than 5 years of experience were 0.039 times less likely to have high level of organizational commitment than others; and respondents with more than 5 years of positional tenure were 1.632 times more likely to have high level of organizational commitment than others.

**Table 3:** Analysis of Respondent’s Work Experience as a Predictors of Organizational Commitment

					*n* = 197
Work Experience	Organizational Commitment		*p*-value ^a^	AOR	95% CI
	Low and Moderate Level	High Level			
	No. (%)	No. (%)			
Positional Tenure					
≤ 5 Years ^b^	110 (72.8)	41 (27.2)			
> 5 Years	30 (65.2)	16 (34.8)	.402	1.632	[0.519, 5.128]
Professional Tenure					
≤ 5 Years ^b^	14 (77.8)	4 (22.2)			
> 5 Years	126 (70.4)	53 (29.6)	.424	0.524	[0.108, 2.553]
Current Organizational Tenure					
≤ 5 Years ^b^	93 (73.2)	34 (26.8)			
> 5 Years	47 (67.1)	23 (32.9)	.044^c^	0.039	[0.098, 0.967]

*Note.* ^a^: *p*-value obtained from binary logistic regression. ^b^ = reference category. ^c^ = *p*-value significant at ≤0.05 level. *AOR* Adjusted Odds Ratio

Table 4 shows that job satisfaction (*p* = .032, AOR: 2.608, CI: 1.087, 6.255) contributed significantly to the prediction of high level of organizational commitment, but contribution of other variables – having economically dependent family members, perceived faculty developmental opportunity, and perceived organizational support were not significant. However, the adjusted odd ratio reveals that the respondents who did not have economically dependent family members, who had a favorable perception towards faculty developmental opportunity, and a high level of perceived organizational support had 2.001, 1.713, and 1.189 times more likely to have high level of organizational commitment than others.

**Table 4:** Respondent’s Having Economically Dependent Family Members, Perceived Faculty Developmental Opportunity, JS and POS as Predictors of OC

					*n* = 197
Variables	Organizational Commitment		*p*-value^a^	AOR	95% CI
	Low and Moderate Level	High Level			
	No. (%)	No. (%)			
Having Economically Dependent Family Members					
Yes^b^	78 (77.2)	23 (22.8)			
No	62 (64.6)	34 (35.4)	.080	2.001	[0.921, 4.345]
Perceived Faculty Developmental Opportunity					
Neutral and Unfavorable Perception^b^	73 (84.9)	13 (15.1)			
Having Favorable Perception	67 (60.4)	44 (39.6)	.270	1.713	[0.658, 4.457]
Job Satisfaction (JS)					
Dissatisfaction and Ambivalence^b^	105 (80.8)	25 (19.2)			
Satisfaction	35 (52.2)	32 (47.8)	.032^c^	2.608	[1.087, 6.255]
Perceived Organizational Support (POS)					
Low^b^	84 (80.0)	21 (20.0)			
High	56 (60.9)	36 (39.1)	.481	1.189	[0.735, 1.922]

*Note.* ^a^ = *p*-value obtained from binary logistic regression. ^b^ = reference category. ^c^ = *p*-value significant at ≤0.05 level. *AOR* Adjusted Odds Ratio

## Discussion

This study reveals that a majority of respondents had a moderate level of organizational commitment (68%) and 29% had high level. The findings of a previous study on effect of organizational climate on organizational commitment among faculty of nursing in Egypt indicated that most faculty had a moderate level of organizational commitment [19]. The findings of the current study are dissimilar with the findings of an empirical investigation of faculty members’ organizational commitment in Saudi Arabia, which revealed that faculty had high level (73.4%) and moderate level (26.0%) of commitment for overall organizational commitment [20]. Another study on assessment of work environment and employee’s commitment in college of nursing in Saudi Arabia also revealed that the respondents had high commitment scores (61.2%), and moderate commitment scores (38.8%) [21]. Similarly, a study on organizational commitment among faculty of an educational institute in India also revealed that there was a high level of organizational commitment among the faculty members of the university [3]. The dissimilarities in results from countries might be due to variation in geography, available opportunities, and facilities in each organization.

Regarding predictors of organizational commitment among respondents, the current study reveals that age did not contribute significantly to the prediction of a high level of organizational commitment. This finding is supported by a previous study on determinants of organizational commitment among the faculty of private tertiary institutions in the Philippines which showed that age did not significantly affect organizational commitment [22]. A similar finding from a study on impact of personal attributes on the commitment level among faculty of educational institutions in Pakistan revealed that age was not a good predictor of commitment [23]. The supportive argument related to association between age and organizational commitment is that older employees may assume that they have accumulated personal capital in the organization such as self-identity, friends and social relationships, seniority or retirement benefits which they would not want to lose by leaving an organization. Therefore, they might be more committed to their organization.

The present study shows that marital status did not contribute significantly to the prediction of high level of organizational commitment. The finding of the current study is inconsistent with the finding of a previous study on organizational commitment and turnover intention among private universities’ employees in Nigeria, which revealed that marital status significantly predicted organizational commitment and turnover intention [8]. The supportive argument related to the association between marital status and organizational commitment is that married employees are more committed, since they have more financial responsibilities to their families. The contradictory result of the current study might be due to the changing life style and equal sharing responsibilities between couples. Both single and married employees might have same level of financial needs to meet the high living expenses and higher demands for a better living standard.

The recent study highlights that higher education in nursing contributed significantly to the prediction of high level of organizational commitment. The finding is consistent with a comparative study on qualification and organizational commitment among the faculty of private universities in Pakistan, which revealed that faculty holding a Master’s degree were more dedicated than those holding MPhil and PhD degrees [7]. The supportive argument for the current study concerning the association between education and organizational commitment is that employees with a higher level of education are likely to leave their current organization. The search for a more fulfilling and dignified job in spite of the type of job, which they are doing, or numbers of years they have put into their current job. The opposite result might occur due to feeling of inadequacy related to opportunity at their current job despite their qualifications.

The current study also revealed that position contributed significantly to the prediction of low to moderate level of organizational commitment. The finding of the current study is consistent a previous study of Saudi Arabia which showed that academic rank was found to be significantly related to organizational commitment [20]. The supportive argument related to association between position and organizational commitment is that at a higher position, opportunities and responsibilities also increase. Therefore, the opportunity and fulfilled responsibilities made employees feel more responsible in decision making and are better integrated into workplace.

The current study shows that permanent type of appointment contributed significantly to the prediction of a high level of organizational commitment. This finding is inconsistent with the finding of a previous study conducted among nurses in Nepal, which did not reveal type of appointment as a predictor of organizational commitment [9]. The supportive argument related to association between type of appointment and organizational commitment is that permanent employees are more committed, since they have a sense of greater job security and job fulfillment.

The current study highlights that respondents with more than 5 years of work experience in current organization were less likely to have a high level of organizational commitment. However, positional tenure and professional tenure did not contribute significantly to the prediction of high level of organizational commitment. A previous study done among nurses in Nepal did not reveal work experience as a predictor of organizational commitment [9]. The supportive argument related to association between work experience and organizational commitment is that employees that have more years of work experience are likely to stay with their existing organization, since they have invested much time and effort, attained seniority and are connected to the organization.

The present study shows that having economically dependent family members did not contribute significantly to the prediction of high level of organizational commitment. This finding is consistent with the previous study conducted among nurses in Nepal, which did not reveal economically dependent family members as a significant predictor of organizational commitment [9]. The supportive argument related to association between having financially dependent family members and organizational commitment is that employees who have economically dependent family members might have higher level of economic needs to meet the high living expenses and basic needs of their dependent members. Therefore, they are more likely to be committed to their organization.

The current study shows that perceived faculty developmental opportunities did not contribute significantly to the prediction of high level of organizational commitment. This finding is similar to a previous study among nurses, which did not reveal staff developmental opportunity as a predictor of organizational commitment [9]. The supportive argument related to association between perceived faculty developmental opportunity and organizational commitment is that each employee might desire to further develop their potential and achieve professional growth and self-actualization. If they feel a favorable perception towards faculty developmental opportunities, they are more likely to be committed.

The present study shows that job satisfaction contributed significantly to the prediction of high level of organizational commitment. The previous study conducted in Jordan showed that job satisfaction was significantly related to faculty members’ commitment [6]. The supportive argument related to association between job satisfaction and organizational commitment is that satisfied employees are more likely to be creative, innovative, motivated for increasing their job performance and are more likely to be committed towards their organization. Therefore, effective measures should be taken by relevant authorities at each university to improve job satisfaction among university nursing faculty and enhance organizational commitment.

The present study demonstrates that perceived organizational support did not contribute significantly to the prediction of high level of organizational commitment. This finding is different from previous findings on structural relationships between organizational commitment, job satisfaction, developmental experiences, work values, organizational support, and person-organization fit among nursing faculty in USA. That study revealed that perceived organizational support positively predicted nurse faculty’s organizational commitment to the academic organization [24]. Another study carried out in Jordan showed that perceived organizational support was significantly related to faculty members’ commitment [6]. The supportive argument related to association between perceived organizational support and organizational commitment is that employees who perceive a high level of organizational support may feel confident and hopeful about their desired job goals and are more likely to be committed towards their organization. Therefore, providing more support to nursing faculty by adopting faculty centered strategies such as taking into consideration of their best interests, valuing their work, and providing help when they face difficulties may enhance organizational commitment.

## Conclusion

In conclusion, university nursing faculty have moderate level of organizational commitment. Higher education in nursing, position, type of appointment, current organizational tenure and job satisfaction are predictors of organizational commitment. Hence, human service organizations must focus on developing strategies to retain experienced employees by offering permanent appointments and provide professional and academic career development tools. They must do more to offer novel faculty developmental opportunities, provide organizational support, and improve job satisfaction. These cumulative actions will foster an environment for enhancing organizational commitment amongst nursing faculty. Effectively, such an ecosystem will reduce turnover, improve quality of teaching, lower absenteeism, improve the student’s satisfaction level, and enhance organizational effectiveness.

### Limitations

The limitations of this study were: the results were derived only from the self-reported techniques based on perceptions of nursing faculty using Likert items. There is a possibility of social desirability and central tendency biases, which may have distorted the true organizational commitment. Moreover, the generalizability of the study’s findings is limited because of the study population, which was based on responses from nursing faculty of different nursing colleges within Kathmandu Valley. To obtain more generalizable results, future investigations should include nursing faculty working outside Kathmandu Valley as well. The findings were drawn from cross-sectional data obtained from self-administered questionnaires and this study had not included other variables that may influence organizational commitment i.e., organizational culture, job involvement, job stress, job insecurity, workplace harassment, organizational communication and so on. Therefore, this study was not able to present definitive conclusions about the direction of causality and reveal the factors, which have long-term effects on organizational commitment. Hence, prospective longitudinal research should be conducted to identify the antecedent and consequences factors associated with organizational commitment among nursing faculty in different colleges affiliated to different universities of Nepal.

However, this study has methodological strengths. It was a large survey including major universities in Nepal with a range of nursing faculty using random sampling technique with precise sample size. In addition, a high response rate (94.3% after eliminating the missing data) strengthens the generalizability of the study findings within the nursing faculty of Kathmandu valley.

## Abbreviations

JSS: Job Satisfaction Survey
OCQ: Organizational Commitment Questionnaire
POS: Perceived Organizational Support
SPSS: Statistical Package for the Social Sciences
